# Discrimination of Olive Oil and Extra-Virgin Olive Oil from Other Vegetable Oils by Targeted and Untargeted HRMS Profiling of Phenolic and Triterpenic Compounds Combined with Chemometrics

**DOI:** 10.3390/ijms24065292

**Published:** 2023-03-10

**Authors:** Elisabeta-Irina Geana, Corina Teodora Ciucure, Irina Mirela Apetrei, Maria Lisa Clodoveo, Constantin Apetrei

**Affiliations:** 1 National Research and Development Institute for Cryogenics and Isotopic Technologies—ICSI, Rm. Valcea, 240050 Râmnicu Vâlcea, Romania; 2 Department of Pharmaceutical Sciences, Medical and Pharmaceutical Research Center, Faculty of Medicine and Pharmacy, “Dunarea de Jos” University of Galati, 800008 Galati, Romania; 3 Interdisciplinary Department of Medicine, University Aldo Moro Bari, 70125 Bari, Italy; 4 Department of Chemistry, Physics and Environment, “Dunarea de Jos” University of Galati, 800008 Galati, Romania

**Keywords:** biomarker, olive oil authentication, seeds and nuts oils, HRMS analysis, multivariate data analysis

## Abstract

Extra-virgin olive oil (EVOO) and virgin olive oil (VOO) are valuable natural products of great economic interest for their producing countries, and therefore, it is necessary to establish methods capable of proving the authenticity of these oils on the market. This work presents a methodology for the discrimination of olive oil and extra-virgin olive oil from other vegetable oils based on targeted and untargeted high-resolution mass spectrometry (HRMS) profiling of phenolic and triterpenic compounds coupled with multivariate statistical analysis of the data. Some phenolic compounds (cinnamic acid, coumaric acids, apigenin, pinocembrin, hydroxytyrosol and maslinic acid), secoiridoids (elenolic acid, ligstroside and oleocanthal) and lignans (pinoresinol and hydroxy and acetoxy derivatives) could be olive oil biomarkers, whereby these compounds are quantified in higher amounts in EVOO compared to other vegetable oils. The principal component analysis (PCA) performed based on the targeted compounds from the oil samples confirmed that cinnamic acid, coumaric acids, apigenin, pinocembrin, hydroxytyrosol and maslinic acid could be considered as tracers for olive oils authentication. The heat map profiles based on the untargeted HRMS data indicate a clear discrimination of the olive oils from the other vegetable oils. The proposed methodology could be extended to the authentication and classification of EVOOs depending on the variety, geographical origin, or adulteration practices.

## 1. Introduction

Among edible oils, virgin olive oil (VOO) and, especially, extra-virgin olive oil (EVOO) present important and outstanding characteristics due to their differentiated sensory qualities (taste and aroma) and high nutritional value, which is associated with their high content of natural antioxidants, such as carotenoids, phytosterols, flavonoids, α-tocopherol and other phenolic compounds [1,2]. The consumption of VOO and EVOO shows numerous health benefits, including lowering of LDL cholesterol, as well as protection against diseases such as cancer, obesity, diabetes, kidney, neurodegenerative and cardiovascular diseases due to their antioxidant, anti-inflammatory, antiviral and antimicrobial properties [3].

Olive oil is the main component of the Mediterranean diet, and is composed of a saponifiable fraction accounting for between 90% and 99%, which contains the fatty acids and tri-acylglycerols that form the largest part of the olive oil, as well as an unsaponifiable fraction accounting for between 0.4% and 5% that contains over 200 different compounds, including phenolic and triterpenic compounds, sterols, hydrocarbons and tocopherols [4]. The phenolic component contributes to the stability of the oil during processing and storage, as well as to the organoleptic and nutritional qualities of the oil [5,6].

Compared to other edible oils, EVOO is unique due to the presence in its composition of phenolic compounds, named biophenols, which can be classified into five main classes: phenolic acids (chlorogenic, caffeic, p-hydroxybenzoic, protocatechuic, vanillic, syringic, p-coumaric and o-coumaric acids), phenolic alcohols (tyrosol (p-hydroxyphenyl ethanol or p-HPEA) and hydroxytyrosol (3,4-dihydroxyphenyl ethanol or 3,4-DHPEA)), secoiridoids (oleuropein, demethyloeuropein, oleuropein aglycone, oleocanthal, elenolic acid, ligstroside, nuzhenid), flavonoids (apigenin-7-glucoside, luteolin-7-rutinoside, luteolin-7-glucoside, luteolin-5-glucoside, quercetin-3-rutinoside) and lignans (acetoxypinoresinol, pinoresinol) [1,7]. In EVOO and VOO, the major components of the phenolic fraction are tyrosol and hydroxytyrosol, including their derivatives (hydroxytyrosol glucoside, hydroxytyrosol acetate, tyrosol acetate), secoiridoids, and secoiridoid derivatives [8]. These biophenols are transferred into olive oils from olive drupes and leaves during the pressing process, and thus represent characteristic biomarkers of olive oils [9,10]. Apart from biophenols, triterpene compounds such as maslinic and oleanolic acids are also characteristic secondary metabolites, being abundant in olive oil and contributing to several biological effects [11,12].

It is important to emphasize that biophenols, and mainly secoiridoids, such as oleuropein aglycone and oleocanthal, are responsible for the organoleptic characteristics of EVOO, especially its bitter and pungent taste. In addition, these compounds contribute to the oxidative stability of VOO and its long shelf life compared to other edible vegetable oils [13,14]. Hydroxytyrosol, oleocanthal, luteolin, tyrosol, vanillin, acetoxypinoresinol and pinoresinol represent olive oil biophenols that possess strong antioxidant activity, and which can act as potential agents for the prevention and treatment of many oxidative stress-related diseases, like cardiovascular and neurodegenerative diseases, cancer and diabetes [15,16].

The concentration and composition of biophenols can be influenced by geographical origin, and variety (mainly the genotype), as well as several agronomic and technological parameters [1,17,18].

Although there is no specific legislation related to these compounds in food, European labeling regulations [19] require that nutrition and health claims be based on scientific information, studies, and the composition of bioactive compounds, including with respect to their qualitative and quantitative characteristics. Despite the great importance of olive polyphenols, their final concentration in the oil is indeed questionable, as the process during oil production can destroy, degrade or simply waste large amounts of valuable secondary metabolites [20].

The analysis of phenolic compounds in olive oils is carried out using chromatographic methods, especially high-performance liquid chromatography (HPLC) coupled with DAD, electrochemical, and MS detections [21,22,23,24]. HRMS analysis offers improved resolution and stability for accurate mass measurements along with accurate targeted and untargeted analysis [25]. Gas chromatography (GC) analysis is less common due to the need to derivatize the sample prior to instrumental detection [26,27]. Additionally, electrochemical sensors, including electronic tongue and noses were used for the quantification of the main phenolic compounds presents in olive oils [28,29,30,31,32].

Isolation of phenolic compounds from the olive oil matrix is generally a prerequisite for any comprehensive analysis scheme, with the resulting extract being uniformly enriched in all compounds of interest and free of interfering matrix components. Due to the differences in molecular size, polarity and stability of the phenolic compounds in olive oil, a crucial step in the analytical procedure for their determination, in terms of their recovery from the matrix, is the identification of a suitable method for the quantitative isolation of the phenolic fraction from olive oil [33]. Liquid–liquid extraction (LLE) and, more recently, solid-phase extraction (SPE) have been used to isolate the so-called “polar fraction”. The solvent system usually applied is aqueous methanol in various proportions [34]. The versatility of the SPE extraction technique has been exploited for the recovery of phenolic compounds from olive oil, and various systems using SPE, either as an isolation step or as a purification step, have been reported in the literature. Some of the suitable adsorbents include alkyl silicones such as C8 or C18 (but incomplete extraction of the phenolic fraction and partial separation of the oil have been reported) [35]. Anion exchange cartridges have also been used to isolate the phenolic fraction from various seed oils, but levels of recovery were low (53–62%) for some components. Promising results were obtained with amino-phase and diol-bond phase SPE cartridges, with high recovery (>90%) of all major olive phenolic compounds being found for the latter [36,37,38].

The aim of this study is to characterize the minor and major biophenols and triterpenic (oleanolic and maslinic acids) compositions of VOO, EVOO and other vegetable oils (walnut, grape seed, pumpkin, linseed, soybean, sesame, palm, hemp, coconut and sunflower oils) using targeted and untargeted UHPLC-HRMS analysis. Principal component analysis (PCA) and Heat Map Analysis (HMA) were performed in order to discriminate different types of vegetable oils and identify specific biomarkers of EVOOs and VOOs as tracers for the olive oils authentication process.

## 2. Results and Discussion

### 2.1. Identification of Phenolic and Triterpenic Compound Biomarkers in the Investigated Vegetable Oils by UHPLC-HRMS

**Figure 1:**
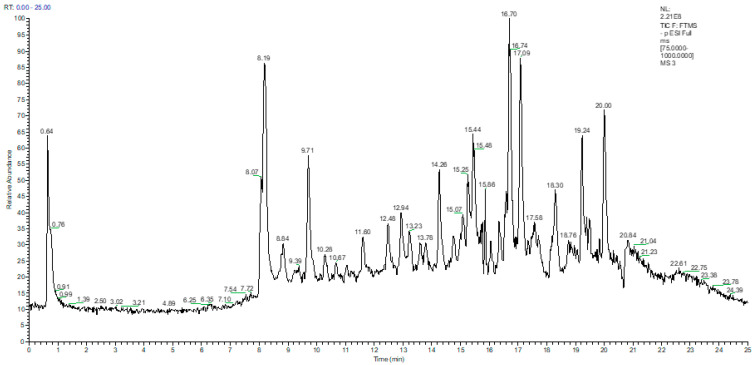
The total ion current (TIC) chromatogram obtained for the separation of targeted phenolic compounds from extra-virgin olive oil liquid extract using UHPLC–MS/MS detection in negative ionization mode.

The identification of the quantified phenolic compounds in the vegetable oils was carried out on the basis of a comparison of the retention times with those of the reference compounds, and through the identification of the molecular ion and the fragments resulting from the ionization in the negative mode (Figure 1 and Table 1). The Total Ion Current (TIC) chromatogram of the EVOO extract in the negative ion mode, covering a scan range between 75 and 1000 *m/z*, is shown in Figure 1, while the extracted chromatograms of the main phenolic and triterpenic compounds quantified in EVOO (the chromatograms were extracted from TIC using a 5 ppm mass accuracy window, negative ion mode, full scan, base peak in the range 75–1000 *m/z*) are illustrated in Figures S1 and S2).

A total of 30 bioactive compounds were simultaneously identified and quantified in comparison to the reference standards, including seven phenolic acids, two phenolic alcohols, 12 flavonoids, two triterpenic compounds, stilbenes (t-resveratrol), plant hormone (abscisic acid), and ellagic acid (a dimeric derivative of gallic acid), as well as specific olive oil biomarkers belonging to the classes of alkaloids (trigonelline), secoiridoids (oleuropein) and caffeoyl phenylethanoid glycoside (verbascoside). The retention time, compound name, formula, and *m/z* values of the adduct ions, as well as the MS/MS fragment ions in negative ESI mode, mass error, and accurate molecular mass, are shown in Table 1.

Untargeted HRMS analysis allows the identification of other bioactive biomarkers and specialized metabolites that occur in vegetable oils, which are also responsible for the particular sensorial and bioactive properties. Data processing analysis was performed using Compound Discoverer software, following a metabolomics working template that included RT alignment, background annotation, the assignment and comparison of fragmentation pattern, and molecular formula prediction based on the automated library and database search for identification purposes, including mzCloud (MS2 fragments), Chemspider, MzVault and Mass List Matches [39].

The most abundant HRMS signals in the EVOO and VOO extracts were those corresponding to a large number of phenolic compounds typical of the olive tree, the subclass secoiridoids, both in their free form and when esterified to form secoiridoid derivatives (such as ligstroside and oleuropein derivatives). The high resolving power of the mass analyzer combined with data processing using Compound Discoverer software allowed the identification of most of these compounds based on the observation of specific and characteristic fragments and/or neutral losses. The extracted chromatograms (using a mass accuracy window of 5 ppm) of the main biophenols (simple phenols and derivatives, flavonoids, secoiridoids and derivatives, lignans) in VOO extracts are presented in Figures S3 and S4. The compound names, molecular formulas, retention times, precursor ion mass and fragment ion data of these compounds are summarized in Table 2.

Phenolic acids, such as syringic, vanillic, homogentisic, homovanillic, sinapic, caffeic and caftaric acids possess strong natural antioxidants properties, and are responsible for a wide range of biological properties and sensory features in virgin olive oil [40], while some derivatives such as hydroxytyrosol glucoside, hydroxytyrosol acetate, tyrosol acetate, syringaldehyde, and 3,4-dihydroxyphenyl glycol that contribute to the health-promoting effects (protection of blood lipids from oxidative stress) are associated with the dietary intake of olive oils [8]. Among flavonoids, luteolin, genistein and daidzein, compounds which exhibit strong antioxidant potential, have been identified in olive oils in a supplementary capacity [41]. 

Secoiridoids such as oleuroside, elenolic acid, ligstroside aglycone, secoiridoid derivatives (oleocanthal or p-HPEA-EDA (Ligstroside aglycone decarboxymethyl dialdehyde form); 3,4-DHPEA-EDA (Dialdehydic decarboxymethyloleuropein aglycone); 3,4-DHPEA-EA (Aldehydic decarboxymethyloleuropein aglycone)), and lignans ((±)-pinoresinol, 8-hydroxypinoresinol, acetoxypinoresinol) represent the major group of phenolic compounds identified in the EVOO and VOO. These compounds have been associated with some remarkable health effects of virgin olive oil intake and contribute to the higher oxidative stability and higher bitterness intensity of EVOO and VOO [42].

### 2.2. Phenolic Compound Composition of the Investigated Vegetable Oils

**Figure 2:**
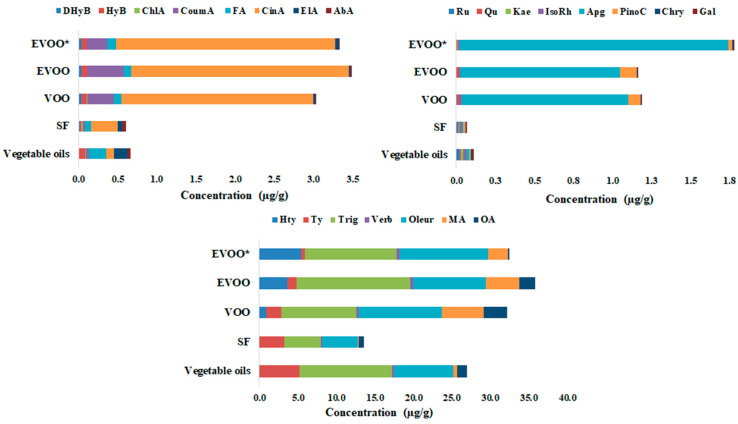
Biophenolic and triterpenic acids profiles of authentic extra-virgin olive oils (EVOO*), extra-virgin olive oils (EVOO) and virgin olive oils (VOO) compared with sunflower oils (SF) other vegetable oils (average values for grape seeds, pumpkin, linseed, sesame, hemp, rape, walnut, palm, rice, almond, coconut and soybean oils).

Targeted analysis confirmed large variations in the contents of some phenolic acids and alcohols, flavonoids, simple secoiridoids and triterpenic acids among the different vegetable oil types, with a range of concentrations being present within each group of oils (EVOO*—authentic extra-virgin oils; EVOO—commercially available extra-virgin oils; VOO—commercially available virgin olive oil; SF—sunflower oils, as well as other vegetable oils including walnut, grape seed, pumpkin, linseed, soybean, sesame, palm, hemp and coconut oils). Among the target bioactive compounds, gallic acid, catechin, epicatechin, naringin, t-resveratrol, hesperidin and galangin were not identified or were quantified in low amounts in the oil extracts. The quantitative data for the different oil types are presented in Table S1 as mean values and standard deviations, and the range of variation for each type of oil category is presented in Figure 2.

The quantitative data for the phenolic compounds in vegetable oils indicate that the main phenolic acids quantified were cinnamic (CinA) and p-coumaric (CoumA) acids in olive oils, and ferulic (FA), ellagic (ElA) and abscisic (abA) acids in the other vegetable oils, while the main flavonoids were apigenin and (Apg) and pinocembrin (PinoC), which are characteristic of olive oils. Tyrosol (Ty) was quantified in higher amounts in other vegetable oils compared with in olive oils, while hydroxytyrosol (HTy) was quantified only in olive oils, with higher amounts being found in extra-virgin olive oils. Trigonelline (Trig) and oleuropein (Oleur), which are specific biomarkers of olive oils, were also quantified in the other vegetable oils. Among the triterpenic acids, maslinic acid (MA) is representative of olive oils, while oleanolic acid (OA) seems to be more specific to other vegetable oils, since authentic extra-virgin olive oils (EVOO*) show low amounts of OA. 

The cinnamic acid contents of EVOO (n.d.–5.08 µg/g) and VOO (0.02–4.83 µg/g) were ten times greater than those of sunflower oils (0.01–1.52 µg/g) and other vegetable oils (0.01–0.37 µg/g) (Table S1), with the reported values being higher compared with reported literature data (n.d.–0.64 µg/g) [43,44]. Additionally, the amounts of p-coumaric acid in EVOO (n.d–0.54 µg/g) and VOO (0.01–0.71 µg/g) were significantly higher than those found in sunflower (0.01–0.09 µg/g) and other vegetable (0.01–0.18 µg/g) oils, while the obtained values were lower than those reported for EVOO from Croatia (0.43–5.16 µg/g) [45] and Spain (0.31–5.77 µg/g) [43] and for VOO (0.03–1.33 µg/g) [44]. Among the quantified flavonoids, apigenin and pinocembrin are characteristic of EVOO and VOO, with values between n.d. and 6.49 µg/g for apigenin and between n.d. and 0.38 µg/g for pinocembrin. The level of apigenin found in the investigated olive oils is comparable with the level reported in the literature [13,43,46,47], while pinocembrin has not been reported in the literature.

Hydroxytyrosol was not quantified in sunflower oils or the other investigated vegetable oils, while the highest amounts were found in EVOO* (authentic EVOO collected from Italian producers) (0.01–24.58 µg/g), followed by commercial EVOO (0.01–10.72 µg/g) and commercial VOO (n.d.–5.38 µg/g). Tyrosol was quantified in higher amounts in the vegetable oils obtained from seeds (n.d.–12.76 µg/g) compared with in olive oils (n.d.–10.39 µg/g). The reported values of hydroxytyrosol are comparable with those reported by Lechhab et al. (0.26–7.81 µg/g) [48], Faghim et al. (5.35–13.42 µg/g) [47], Klisović et al. (4.25–6.60 µg/g) [49], and Miho et al. (0.71–2.7 µg/g) [13], but lower than those reported by Di Stefano and Melilli et al. (34.50 µg/g) [46] and Becerra-Herrera et al. (13.03–72.71 µg/g) [43]. The quantity of tyrosol found in the investigated olive oils was similar to that reported by Faghim et al. (9.52–10.65 µg/g) [47], Klisović et al. (4.25–6.60 µg/g) [49] and Arslan et al. (5.83–9.68 µg/g) [44].

The content of maslinic acid in olive oils (0.24–18.73 µg/g) was significantly higher compared with the other vegetable oils (n.d.–4.53 µg/g), while oleanolic acid was quantified in higher amounts in commercial VOO (0.07–16.07 µg/g) and EVOO (0.69–6.15 µg/g), as well as in grape seed oil (15.45 µg/g) (Table S1).

Oleuropein, a major polyphenolic compound enriched in olive oil from leaves of the olive tree [50], was quantified in lower amounts in the studied authentic (0.81–18.81 µg/g) and commercial (0.30–21.81 µg/g) EVOO and VOO (0.81–33.0 µg/g) than in the reported literature data (40.71–248.1 µg/g) [46,47,49]. Surprisingly, oleuropein was also quantified in low quantities in some of the commercial vegetable oils (e.g., sunflower, rape, sesame, rice and almond), indicating a possible supplementation of these oils with olive leaf extracts in order to increase their oxidative stability and nutraceutical potential [51].

From the target HRMS analysis of biophenols and triterpenic acids in olive oils and other types of vegetable oil, it can be concluded that cinnamic and p-coumaric acids, apigenin, pinocembrin, hydroxytyrosol and maslinic acid can be considered to be specific olive oil biomarkers, with these being quantified in higher amounts in EVOO (both authentic and commercial), and commercial VOO compared with sunflower and other seed oils (walnut, grape seed, pumpkin, linseed, soybean, sesame, palm, hemp and coconut).

### 2.3. Discrimination of Olive Oils from Other Vegetable Oils Based on Targeted and Untargeted HRMS Analysis of Phenolic Compounds and Triterpenic Acids Biomarkers

Unsupervised multivariate methods including PCA and HMA were used to reduce the dimensionality of the original data matrix, while retaining the maximum amount of variability, which allows differentiation between different oil types based on target HRMS data of biophenols and triterpenic acids, but also based on untargeted HRMS profiling of the bioactive compound from oils. It was therefore possible to explain the differences between the investigated olive oils (EVOO*—authentic extra-virgin olive oils obtained from Italian producers, but also commercial EVOO and VOO) and other vegetable oils obtained from seeds (sunflower, grape, pumpkin, linseed, sesame, hemp), rape and nuts (walnut, palm, rice, almond, coconut and soybean), and to determine which variables contributed the most regarding such differences.

**Figure 3:**
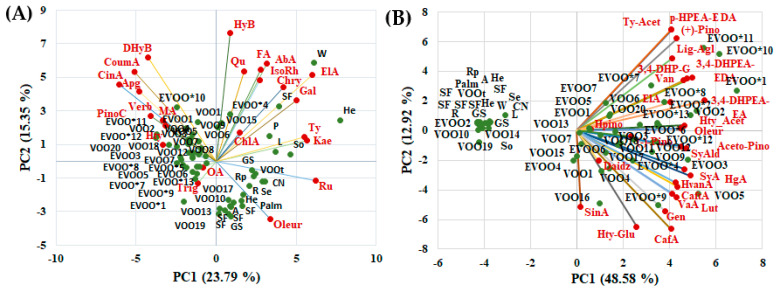
PCA results (scores and loading biplots) of different vegetable oils based on: (**A**) targeted HRMS analysis of phenolic compounds and triterpenic acids biomarkers and (**B**) untargeted HRMS screening analysis. (EVOO*—authentic extra-virgin olive oils; EVOO—commercial extra-virgin olive oil; VOO—commercial virgin olive oil; SF—sunflower oil; GS—grape seeds oil; P—pumpkin oil; L—linseed oil; Se—sesame oil; He—hemp oil; Rp—rape oil; W—walnut oil; P—palm oil; R—rice oil; A—almond oil; CN—coconut oil; and So—soybean oil).

First, principal component analysis was performed as an exploratory analysis of data related to the content of phenolic and triterpenic compounds obtained from targeted HRMS analysis and semiquantitative data obtained from the untargeted HRMS screening analysis (the area corresponding to the main representative signals in the HRMS spectra). The distribution of vegetable oils in the PC1-PC2 score plot is presented in Figure 3. The first two components of the PCA model accounted for 39% of variance based on targeted analysis (Figure 3A) and 62% for untargeted screening analysis (Figure 3B), with a higher contribution brought by PC1 when compared to PC2, in both cases.

PCA indicated a clear discrimination between olive oils (EVOO*, EVOO and VOO) and other vegetable oils, but no discrimination was observed between the different oils from seeds and nuts, probably due to the small number of samples for each type of oil and the very large variety of investigated vegetable oils (SF, GS, P, L, Se, He, Rp, W, Palm, R, A, CN and So). On the basis of the targeted analysis (Figure 3A), the EVOO and VOO oil samples were grouped on the left side of the PC1-PC2 score plot, being characterized by specific biomarkers, such as cinnamic (CinA), p-coumaric (CoumA) and 3,4-Dihydroxybenzoic (DHyB) acids, apigenin (Apg), pinocembrin (PinoC), maslinic acid (MA), hydroxytyrosol (HTy) and trigonelline (Trig), while the other vegetable oils were distributed on the right side of the PC1-PC2 score plot. Phenolic compounds such as ferulic (FA), hydroxybenzoic (HyB), elagic (ElA), abscisic (AbA) and chlorogenic (ChlA) acids, quercetin (Qu), isorhamnetin (IsoRh), kaempferol (Kae), rutin (Ru) and tyrosol (Ty) represent the specific biomarkers for oils from seeds and nuts. Some EVOO and VOO oils were grouped together with the other vegetable oils, probably due to the olive variety (in the case of the EVOO*4 sample) or other manufacturing processes (i.e., the processing of the cakes remaining after pressing the olives, in the case of the VOOt sample), as well as the possible adulteration of the commercial olive oil with other vegetable oils, such as sunflower oil (in the case of sample VOO15). Additionally, in the case of untargeted HRMS semiquantitative data, the score plot indicates a clear discrimination of the olive oils from the other vegetable oils (Figure 3B), and practically all of the considered variables are located on the right side of the graph, together with the olive oil samples, suggesting that these variables are representative for the purposes of olive oil traceability. Thus, simple phenols (syringic, vanillic, homogentisic, homovanillic, sinapic caffeic and caftaric acids, and vanilin) and derivatives (3,4-dihydroxyphenyl glycol, hydroxytyrosol glucoside, hydroxytyrosol acetate, syringaldehyde, and tyrosol acetate), flavonoids (luteolin, genistein, and daidzein), secoiridoids (oleuroside, elenolic acid, and ligstroside aglycone) and derivatives (p-HPEA-EDA (Ligstroside aglycone decarboxymethyl dialdehyde form) or oleocanthal; 3,4-DHPEA-EDA (Dialdehydic decarboxymethyloleuropein aglycone); 3,4-DHPEA-EA (Aldehydic decarboxymethyloleuropein aglycone)) and lignans ((±)-pinoresinol, 8-hydroxypinoresinol, and acetoxypinoresinol) are characteristic of olive oils, being absent or quantified in very low amounts in the other vegetable oils

The PCA performed based on the targeted compounds from the oil samples confirmed some specific biomarkers of olive oils that are also highlighted in Figure 2, suggesting that cinnamic (CinA) and p-coumaric (CoumA) acids, apigenin (Apg), pinocembrin (PinoC), maslinic acid (MA) and hydroxytyrosol (HTy) can be considered to be tracers for olive oil authentication.

**Figure 4:**
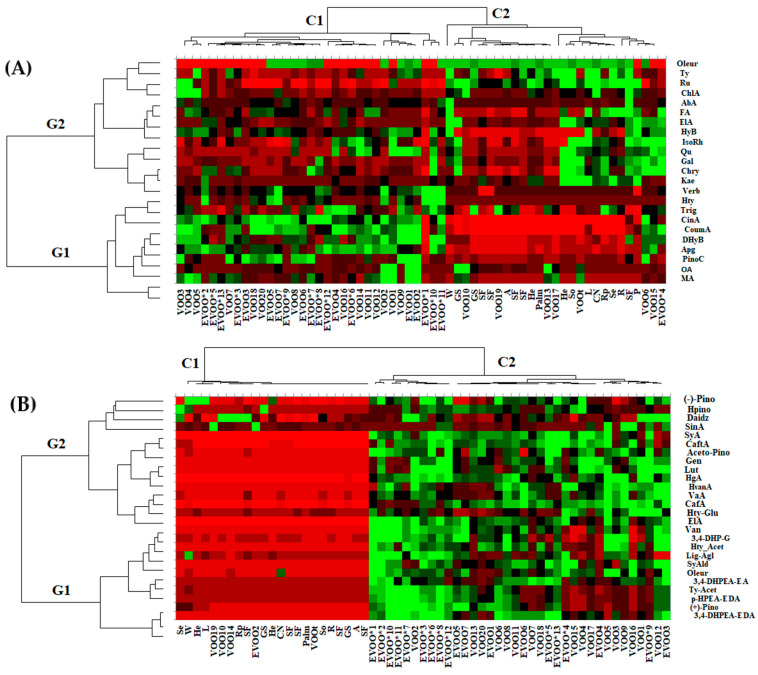
The heat map of discriminant features according to the different type of vegetable oils (red and green cells correspond to low and high compounds levels, respectively. Columns are oil samples and rows are compounds colored by behavior distribution among different oil types). (**A**) Targeted HRMS analysis of phenolic compounds and triterpenic acids biomarkers and (**B**) untargeted HRMS screening analysis. Color scale: red (higher values) to green (lower values) through black. (EVOO*—authentic extra-virgin olive oils; EVOO—commercial extra-virgin olive oil; VOO—commercial virgin olive oil; SF—sunflower oil; GS—grape seed oil; P—pumpkin oil; L—linseed oil; Se—sesame oil; He—hemp oil; Rp—rape oil; W—walnut oil; P—palm oil; R—rice oil; A—almond oil; CN—coconut oil; and So—soybean oil).

In order to extract as much information as possible from the acquired data, both targeted data (quantitative data of the main phenolic compounds and triterpenic acids in the investigated oil types) and untargeted data (referring to the greater number of bioactive compounds present in the oils, but with semi-quantitative data represented by the area under the peak) were used to generate the heat map profiles (Figure 4).

As can be seen in Figure 4A, based on targeted data, the oil samples under study were clustered in two main clusters, with cluster C1 corresponding to authentic and commercial EVOO and the majority of the commercial VOO, while cluster C2 conrresponds to the oils obtained from seeds and nuts (SF, GS, P, L, Se, He, Rp, W, Palm, R, A, CN and So), as well as some commercial VOO and one commercial EVOO, the authenticity of which could be questionable. Additionally, the quantified variables were grouped in two main clusters: G1, which groups the variables representative for olive oils (hydroxytyrosol (HTy), trigonelline (Trig), cinnamic (CinA), p-coumaric (CoumA) and 3,4-dyhydrobybenzoic (DhyB) acids, apigenin (Apg), pinocembrin (PinoC), maslinic and oleanolic acids), and cluster G2, which groups the phenolic compounds representative for seeds and nuts oils.

The heat map profiles developed on the basis of the untargeted HRMS data of the investigated oil types indicate a clear differentiation of the olive oils (EVOO*, EVOO and VOO) (cluster C2) from the other oils from seeds and nuts (cluster C1) which are presented in red color (representing low levels of the specific biomarkers) (Figure 4B). The semiquantitative variables that are representative for olive oils were grouped into two clusters, with G1 representing the specific biomarkers positively correlated with olive oils, while the cluster G2 groups the specific biomarkers negatively correlated with olive oils in the PCA analysis (Figure 3B).

## 3. Materials and Methods

### 3.1. Chemicals

All chemicals and solvents were obtained from Merck Co. (Darmstadt, Germany), and were of HPLC or analytical grade (>99%) quality. Analytical standards (gallic, abscisic, p-cumaric, cafeic, clorogenic, ferulic, elagic, vanilic, 4-hydroxibenzoic, 3,4-dihidroxibenzoic, t-cinamic and syringic acids, (+)-catechin, (−)–epicatechin, rutin, naringin, hesperidin, quercetin, kaempferol, izorhamnetin, chrysin, pinocembrin, apigenin, galangin, t-resveratrol) were purchased from Sigma-Aldrich (Steinheim, Germany and St. Louis, MO, USA), Merck Co. (Darmstadt, Germany) or HWI group, (Ruelzheim, Germany). 

### 3.2. Sample Collection

The vegetable oils investigated in this study were: extra-virgin olive oils from local producers in Italy (EVOO*) (n = 13), commercially available extra-virgin olive oils (EVOO) (n = 7) and commercially available virgin olive oils (VOO) (n = 20), one virgin olive oil obtained from olive cakes (VOOt), as well as other types of commercially available vegetable oils, such as sunflower (SF) (n = 5), grape seeds (GS) (n = 2), hemp (He) (n = 2) and a sample of each type of oil for pumpkin (P), linseed (L), sesame (Se), hemp (He), rape (Rp), walnut (W), palm (Palm), rice (R), almond (A), coconut (CN) and soybean (So). The details regarding the olive varieties and the production process of the oils are unknown.

### 3.3. Extraction Protocols

To isolate the minor phenolic fraction of the olive oils, we used the method proposed by the International Olive Council (COI/T.20/DocNo 29, November 2009). Briefly, the protocol combines olive oil extraction with methanol/water (80/20), ultrasonic bath for 15 min at ambient temperature, and centrifugation at 5000 rpm for 25 min. After that, an aliquot of the supernatant phase is filtered through a 1 mL plastic syringe using 0.45 μm nylon syringe filters (Membrane Solutions, LLC, Auburn, WA, USA) before the injection into the chromatographic system. The isolation of major phenolic compounds from oils was performed by SPE extraction using 500 mg/6 mL NH_2_ SPE cartridges (55 µm, 70 Å) (Phenomenex, Torrance, CA, USA) and a vacuum elution system (Vaccum Manifold, Varian, Dorfen, Germany). The SPE extraction protocol involves conditioning the SPE cartridges with 6 mL of methanol and 6 mL of hexane, followed by the addition of the sample solution (2.5 g of oil in 6 mL of hexane), penetration into the cartridge, washing the cartridges with 3 × 3 mL hexane and eluting the compounds from the cartridge with 10 mL methanol. The resulting sample solution is concentrated to dryness in a stream of nitrogen using a Biotage LV Multivapor (Charlotte, NC, USA), after which the extract was reconstituted with 1 mL methanol:ultrapure water = 80:20 solution and filtered through 0.45 μm nylon syringe filters. The phenolic extracts were stored at −20 °C until UHPLC-HRMS analysis. The same procedures were also applied for other oils under study. 

### 3.4. Targeted and Untargeted UHPLC-HRMS Analysis of Minor and Major Biophenols and Triterpenic Compounds

The targeted analysis of minor and major biophenols and triterpenic compounds (oleanolic and maslinic acids) from VOO, EVOO and other types of vegetable oils and untargeted HRMS analysis were performed by UHPLC-ESI/HRMS (ultra-high-performance liquid chromatography–electrospray ionization–tandem mass spectrometry) using a high-resolution Q Exactive mass spectrometer™ Focus Hybrid Quadrupole–OrbiTrap equipped with HESI, coupled to a high-performance liquid chromatograph UltiMate 3000 UHPLC (Thermo Fisher Scientific, Waltham, MA, USA). The chromatographic separation was performed on a Kinetex C18 column (100 × 2.1 mm, 1.7 µm particle diameter) at 30 °C, under a gradient elution of two mobile phases, A (water with 0.1% formic acid) and B (methanol with 0.1% formic acid), at a flow rate between 0.3 and 0.4 mL/min, as presented in a previous paper [38]. Full scan data in negative mode covering a scan range of *m/z* 75–1000 for minor phenolic compounds and *m/z* 75–1000 for major phenolic compounds and triterpenic compounds were acquired at a resolving power of 70,000 FWHM at *m/z* 200, while variable data-independent analysis MS2 (vDIA) was performed at a resolution of 35,000, with isolation windows and scan ranges being set as follow: 75–205 *m/z*, 195–305 *m/z*, 295–405 *m/z*, 395–505 *m/z* and 495–1000 *m/z*. Nitrogen was used as collision gas and auxiliary gas at a flow rate of 11 and 48 arbitrary units, respectively. The applied voltage was 2.5 kV in the case of minor phenolic compounds and 3.0 kV for major phenolic compounds and triterpenic compounds, and the capillary temperature was 320 °C. The energy of the collision cell was set at 30 eV for minor phenolic compounds and 35 eV for major phenolic compounds. The data were purchased and processed using the Xcalibur software package (Version 4.1) (Thermo Fisher Scientific). The quantification was performed based on external calibration curves covering the concentration range between 25–1750 μg/L for each of the minor phenolic compounds (phenolic acids and flavonoids) and between 25–1000 μg/L for major phenolic compounds, by serial dilution with methanol from the standard mixture of concentration 10 mg/L. The coefficient of linearity ranged from 0.9833 to 0.9996, while the detection limit of the methods, calculated based on a signal to noise ratio of 3:1, ranged between 0.01 and 0.5 µg/mL. The quantitative results for each individual target analyte are expressed as µg/g of oil. To evaluate the performance of the analysis method, the matrix effect was investigated by enriching the oil samples with known concentrations of the standard solution at a concentration level of 100 ng/mL, followed by the analysis of the resulting samples and the estimation of the recovery percentage, with the obtained recoveries being between 75 and 98%. Compound Discoverer software (v. 2.1) using an untargeted metabolomics working template combined with internet database of accurate MS data, ChemSpider (www.chemspider.com, 25 January 2023) and available literature were used as a reference library to identify compounds of interest.

### 3.5. Data Analysis

All the analyses were performed in duplicate. Statistical differences between VOO, EVOO, and different vegetable oils were tested using Pearson’s correlation test at a 0.05 significance level. Principal component analysis (PCA) and heat map analysis were carried out using a data matrix including 57 rows corresponding to the investigated oil samples and 23 phenolic variables resulting from the target HRMS analysis in order to discriminate between different oil types. A Kaiser–Meyer–Olkin (KMO) test was performed in order to test the sampling adequacy, and only variables with values higher than 0.6 were considered. All the data analyses were performed using Microsoft Excel 2010 (Microsoft, Redmond, WA, USA) and XLSTAT Addinsoft version 15.5.03.3707 (Addinsoft, New York, NY, USA).

## 4. Conclusions

This work demonstrates that high-performance liquid chromatography–high-resolution mass spectrometry (UHPLC-HRMS) fingerprinting of bioactive compounds from different types of vegetable oils coupled with multivariate data analysis enables the discrimination of extra-virgin olive oils (EVOO) and virgin olive oils (VOO) from oils obtained from other seeds and nuts and the identification of specific biomarkers of olive oils. Both targeted and untargeted HRMS analysis indicate that EVOO and VOO represent highly complex mixtures of different classes of biophenols, including simple phenols and derivatives, flavonoids, secoiridoids and derivatives, and lignans, as well as triterpenic compounds, making it possible to differentiate them from other types of vegetable oil. 

Principal component analysis (PCA) and heat map analysis were applied to both targeted and untargeted HRMS approaches of different types of vegetable oil, enabling a clear discrimination of EVOO and VOO from other types of oils obtained from seeds and nuts. Most commercially available EVOO and VOO were grouped together with authentic extra-virgin olive oils (EVOO*), thus indicating their authenticity. Cinnamic and p-coumaric acids, apigenin, pinocembrin, maslinic acid, and hydroxytyrosol, as well as simple secoiridoids and derivatives (such as elenolic acid, ligstroside and oleocanthal) and lignans (pinoresinol and hydroxy and acetoxy derivatives), are representative biomarkers of olive oils and can be considered to be tracers for the purposes of olive oil authentication. Further experiments will be performed in order to validate the proposed methodology for the authentication of olive oils.

## Figures and Tables

**Table 1:** The identification of minor and major phenolic compounds and triterpenic compounds in vegetable oils using UHPLC-HRMS with structures confirmed by comparison with reference standards.

No	Compound	Retention Time [min]	Formula	Exact Mass	Accurate Mass [M − H]^−^	Experimental Adduct Ion (*m/z*)	Mass Fragments
**Phenolic acids**							
1	Gallic acid	0.68	C_7_H_6_O_5_	170.0215	169.0142	169.0133	125.0231
2	3,4-Dihydroxybenzoic acid	1.59	C_7_H_6_O_4_	154.0266	153.0193	153.0184	109.0281
3	4-Hydroxybenzoic acid	5.40	C_7_H_6_O_3_	138.0316	137.0243	137.0233	118.9650, 96.9588, 71.0124
4	t-Ferulic acid	8.83	C_10_H_10_O_4_	194.0579	193.0506	193.0499	178.0262, 134.0361
5	Chlorogenic acid	7.55	C_16_H_18_O_9_	354.0950	353.0877	353.0880	191.0553
6	Cinnamic acid	10.45	C_9_H_8_O_2_	148.0524	147.0451	147.0442	119.0489, 103.0387
7	p-Coumaric acid	8.59	C_9_H_8_O_3_	164.0473	163.0400	163.0390	119.0489
**Phenolic alcohols**							
8	Hydroxytyrosol	4.39	C_8_H_10_O_3_	154.0629	153.0551	153.0547	123.0438
9	Tyrosol	9.13	C_8_H_10_O_2_	138.068	137.0602	137.0596	79.9560, 95.9510, 118.9651
**Flavonoids**							
10	Catechin	7.57	C_15_H_14_O_6_	290.0790	289.0717	289.0719	109.0282, 123.0349, 125.0232, 137.0232, 151.0390, 203.0708
11	Epicatechin	8.05	C_15_H_14_O_6_	290.0790	289.0717		
12	Rutin	9.43	C_27_H_30_O_16_	610.1533	609.1460	609.1473	301.0352, 300.0276
13	Naringin	9.25	C_27_H_32_O_14_	580.1791	579.1718	579.1718	363.0721
14	Hesperidin	9.37	C_28_H_34_O_15_	610.1897	609.1824	609.1828	377.0876
15	Quercetin	10.74	C_15_H_10_O_7_	302.2357	301.0354	301.0356	151.0226, 178.9977, 121.0282, 107.0125
16	Isorhamnetin	11.80	C_16_H_12_O_7_	316.0582	315.0509	315.0515	300.0277
17	Kaempferol	11.62	C_15_H_10_O_6_	286.0477	285.0404	285.0406	151.0389, 117.0180
18	Apigenin	11.86	C_15_H_10_O_5_	270.0528	269.0455	269.0455	117.0333, 151.0027, 107.0126
19	Pinocembrin	12.70	C_15_H_12_O_4_	256.0735	255.0662	255.0663	213.0551, 151.0026, 107.0125
21	Chrysin	13.52	C_15_H_10_O_4_	254.0579	253.0506	253.0505	143.0491, 145.0284, 107.0125, 209.0603, 63.0226, 65.0019
22	Galangin	13.77	C_15_H_10_O_5_	270.0528	269.0455	269.0455	169.0650, 143.0491
**Triterpenic compounds**							
23	Oleanolic acid	19.27	C_30_H_48_O_3_	456.3603	455.3525	455.3535	455.3532, 311.0686, 307.1949, 353.2003, 325.1843
24	Maslinic acid	18.09	C_30_H_4_8O_4_	472.3552	471.3474	471.3485	471.3478, 472.3513
**Other compounds**							
25	t-Resveratrol	9.55	C_14_H_12_O_3_	228.0786	227.0713	227.0707	185.0813, 143.0337
26	Ellagic acid	9.66	C_14_H_6_O_8_	302.0062	300.9989	300.9993	300.9990
27	Abscisic acid	10.04	C_15_H_20_O_4_	264.1361	263.1288	263.1290	179.9803, 191.9454
**Specific olive oil biomarkers**							
28	Trigonelline	7.29	C_7_H_7_NO_2_	137.0476	136.0398	136.0393	59.0124
29	Verbascoside	8.85	C_29_H_36_O_15_	624.2054	623.1976	623.1990	623.1992
30	Oleuropein	11.35	C_25_H_32_O_13_	540.1842	539.1764	377.1241	377.1241, 307.0822, 275.0925

**Table 2:** Identification of biophenols in VOO and EVOO extracts by untargeted UHPLC-HRMS analysis of deprotonated precursors and fragment ions of specific components combined with data processing using Compound Discoverer software.

No	Compound	Retention Time [min]	Formula	Exact Mass	Accurate Mass [M − H]^−^	Experimental Adduct Ion (*m/z*)	Mass Fragments
**Simple phenols & derivatives**							
1	3,4-Dihydroxyphenyl glycol	1.12	C_8_H_10_O_4_	170.0579	169.0506	169.0498	72.9917
2	Caftaric acid	2.32	C_13_H_12_O_9_	312.0481	311.0408	311.0386	121.0283, 135.0441,
3	Vanillic acid	3.52	C_8_H_8_O_4_	168.0422	167.0349	167.0343	167.0344, 123.0446, 107.0133
4	Vanillin	4.85	C_8_H_8_O_3_	152.0473	151.0400	151.0391	151.0393, 136.0157, 108.0204
5	Homogentisic acid	6.32	C_8_H_8_O_4_	168.0422	167.0349	167.034	109.0283, 149.0239, 121.0283, 107.0133
6	Sinapic acid	6.47	C_11_H_12_O_5_	224.0684	223.0611	223.0585	137.0234, 111.0076, 95.0490, 69.0332
7	Hydroxytyrosol glucoside	6.62	C_14_H_20_O_8_	316.1158	315.1085	315.1083	185.0815, 157.8522
8	Caffeic acid	7.45	C_9_H_8_O_4_	180.0422	179.0349	179.0342	179.0345, 135.0441
9	Syringic acid	7.67	C_9_H_10_O_5_	198.0528	197.0455	197.045	197.0450, 153.0552, 137.0239
10	Hydroxytyrosol acetate	8.3	C_10_H_12_O_4_	196.0735	195.0662	195.0657	195.0657, 59.0168
11	Syringaldehyde	9.13	C_9_H_10_O_4_	182.0579	181.0506	181.0500	137.0598, 95.0489,
12	Homovanillic acid	9.41	C_9_H_10_O_4_	182.0579	181.0506	181.0500	137.0598, 109.0647, 111.0075
13	Tyrosol acetate	9.88	C_10_H_12_O_3_	180.0786	179.0713	179.0707	179.0708, 137.0603, 119.0497, 59.0133,
**Flavonoids**							
14	Luteolin	10.99	C_15_H_10_O_6_	286.0477	285.0404	285.0408	285.0408, 181.0500, 137.0598
15	Genistein	11.75	C_15_H_10_O_5_	270.0528	269.0455	269.0458	269.0458, 117.0334, 151.0027
16	Daidzein	13.33	C_15_H_10_O_4_	254.0579	253.0506	253.051	146.9602, 174.9554, 110.9746
**Secoiridoids and derivatives**							
17	Oleuroside	8.83	C_25_H_32_O_13_	540.1842	539.1769	539.1764	139.0027, 95.0490
18	Elenolic acid	8.92	C_11_H_14_O_6_	242.0790	241.0717	241.0716	139.0026, 111.0075, 68.9968, 67.0167
19	Ligstroside aglycone	9.63	C_19_H_22_O_7_	362.1365	361.1292	361.1301	341.1011, 221.0429, 181.0500
20	p-HPEA-EDA (Ligstroside aglycone decarboxymethyl dialdehyde form)/Oleocanthal	9.88	C_17_H_20_O_5_	304.1310	303.1237	303.1241	181.0500, 137.0598, 111.0076, 95.0498
21	3,4-DHPEA-EDA (Dialdehydic decarboxymethyloleuropein aglycone	9.96	C_17_H_20_O_6_	320.1259	319.1186	319.119	221.0429, 111.0076, 85.0282
22	3,4-DHPEA-EA (Aldehydic decarboxymethyloleuropein aglycone)	11.35	C_19_H_22_O_8_	378.1314	377.1241	377.1247	181.0500, 137.0598, 109.0647
**Lignans**							
23	(±)-Pinoresinol	9.90/ 10.47	C_20_H_22_O_6_	358.1416	357.1343	357.1349/ 357.1352	151.0392, 137.0598, 123.0440
24	8-Hydroxypinoresinol	9.22	C_20_H_22_O_7_	374.1365	373.1292	373.1297	149.0235, 123.0440, 127.0390, 181.0500
25	Acetoxypinoresinol	10.02	C_22_H_24_O_8_	416.1471	415.1398	415.1405	111.0076, 221.0429, 85.0282

## Data Availability

Not applicable.

## Supplementary Materials

The following supporting information can be downloaded at: https://www.mdpi.com/article/10.3390/ijms24065292/s1.

## References

[1] Kritikou E., Kalogiouri N.P., Kostakis M., Kanakis D.C., Martakos I., Lazarou C., Pentogennis M., Thomaidis N.S. (2021). Geographical Characterization of Olive Oils from the North Aegean Region Based on the Analysis of Biophenols with UHPLC-QTOF-MS. Foods.

[2] Lozano-Castellón J., López-Yerena A., Domínguez-López I., Siscart-Serra A., Fraga N., Sámano S., López-Sabater C., Lamuela-Raventós R.M., Vallverdú-Queralt A., Pérez M. (2022). Extra virgin olive oil: A comprehensive review of efforts to ensure its authenticity, traceability, and safety. Compr. Rev. Food Sci. Food Saf..

[3] Gaforio J.J., Visioli F., Alarcón-De-la-lastra C., Castañer O., Delgado-Rodríguez M., Fitó M., Hernández A.F., Huertas J.R., Martínez-González M.A., Menendez J.A. (2019). Virgin Olive Oil and Health: Summary of the III International Conference on Virgin Olive Oil and Health Consensus Report, JAEN (Spain) 2018. Nutrients.

[4] Luque-Muñoz A., Tapia R., Haidour A., Justicia J., Cuerva J.M. (2019). Direct determination of phenolic secoiridoids in olive oil by ultra-high performance liquid chromatography-triple quadruple mass spectrometry analysis. Sci. Rep..

[5] Gatt L., Lia F., Zammit-Mangion M., Thorpe S.J., Schembri-Wismayer P. (2021). First Profile of Phenolic Compounds from Maltese Extra Virgin Olive Oils Using Liquid-Liquid Extraction and Liquid Chromatography-Mass Spectrometry. J. Oleo Sci..

[6] Alarcón Flores M.I., Romero-González R., Garrido Frenich A., Martínez Vidal J.L. (2012). Analysis of phenolic compounds in olive oil by solid-phase extraction and ultra high performance liquid chromatography–tandem mass spectrometry. Food Chem..

[7] Ocakoglu D., Tokatli F., Ozen B., Korel F. (2009). Distribution of simple phenols, phenolic acids and flavonoids in Turkish monovarietal extra virgin olive oils for two harvest years. Food Chem..

[8] Lammi C., Mulinacci N., Cecchi L., Bellumori M., Bollati C., Bartolomei M., Franchini C., Clodoveo M.L., Corbo F., Arnoldi A. (2020). Virgin Olive Oil Extracts Reduce Oxidative Stress and Modulate Cholesterol Metabolism: Comparison between Oils Obtained with Traditional and Innovative Processes. Antioxidants.

[9] Ansari M., Kazemipour M., Fathi S. (2011). Development of a simple green extraction procedure and HPLC method for determination of oleuropein in olive leaf extract applied to a multi-source comparative study. J. Iran. Chem. Soc..

[10] Losito I., Abbattista R., De Ceglie C., Castellaneta A., Calvano C.D., Cataldi T.R.I. (2021). Bioactive Secoiridoids in Italian Extra-Virgin Olive Oils: Impact of Olive Plant Cultivars, Cultivation Regions and Processing. Molecules.

[11] Kanakis P., Termentzi A., Michel T., Gikas E., Halabalaki M., Skaltsounis A.L. (2013). From olive drupes to olive OilAn HPLC-orbitrap-based qualitative and quantitative exploration of olive key metabolites. Planta Med..

[12] Criado-Navarro I., Ledesma-Escobar C.A., Parrado-Martínez M.J., Marchal-López R.M., Olmo-Peinado J.M., Espejo-Calvo J.A., Priego-Capote F. (2022). Monitoring the partition of bioactive compounds in the extraction of extra virgin olive oil. LWT.

[13] Miho H., Díez C.M., Mena-Bravo A., Sánchez de Medina V., Moral J., Melliou E., Magiatis P., Rallo L., Barranco D., Priego-Capote F. (2018). Cultivar influence on variability in olive oil phenolic profiles determined through an extensive germplasm survey. Food Chem..

[14] Sánchez De Medina V., Priego-Capote F., De Castro M.D.L. (2015). Characterization of monovarietal virgin olive oils by phenols profiling. Talanta.

[15] Basdeki E., Salis C., Hagidimitriou M. (2020). The effects of Mediterranean diet and EVOO consumption in relation to human health. Not. Sci. Biol..

[16] Hashmi M.A., Khan A., Hanif M., Farooq U., Perveen S. (2015). Traditional uses, phytochemistry, and pharmacology of olea europaea (olive). Evidence-based Complement. Evid. -Based Complement. Altern. Med..

[17] Negro C., Aprile A., Luvisi A., Nicolì F., Nutricati E., Vergine M., Miceli A., Blando F., Sabella E., De Bellis L. (2019). Phenolic Profile and Antioxidant Activity of Italian Monovarietal Extra Virgin Olive Oils. Antioxidants.

[18] Papastavropoulou K., Pasias I.N., Dotsika E., Oz E., Oz F., Proestos C. (2022). Separation and Determination of Biophenols in Olive Oil Samples Based on the Official Method of the International Olive Council and Commission Regulation (EU) No. 432/2012. Separations.

[19] European Commission (2006). Regulation (Ec) No 1924/2006 of the European Parliament and of the Council of 20 December 2006 on Nutrition and Health Claims Made on Foods.

[20] Tapia-Quirós P., Montenegro-Landívar M.F., Reig M., Vecino X., Cortina J.L., Saurina J., Granados M. (2022). Recovery of Polyphenols from Agri-Food By-Products: The Olive Oil and Winery Industries Cases. Foods.

[21] Tasioula-Margari M., Tsabolatidou E., Segura-Carretero A., Arrá Ez-Romá D. (2015). Extraction, Separation, and Identification of Phenolic Compounds in Virgin Olive Oil by HPLC-DAD and HPLC-MS. Antioxidants.

[22] Trombetta D., Smeriglio A., Marcoccia D., Giofrè S., Toscano G., Mazzotti F., Giovanazzi A., Lorenzetti S. (2017). Analytical Evaluation and Antioxidant Properties of Some Secondary Metabolites in Northern Italian Mono- and Multi-Varietal Extra Virgin Olive Oils (EVOOs) from Early and Late Harvested Olives. Int. J. Mol. Sci..

[23] Zhu Z., Li X., Zhang Y., Wang J., Dai F., Wang W. (2023). Profiling of phenolic compounds in domestic and imported extra virgin olive oils in China by high performance liquid chromatography-electrochemical detection. LWT.

[24] Giacomino A., Inaudi P., Silletta G., Diana A., Bertinetti S., Gaggero E., Malandrino M., Stilo F., Abollino O. (2023). Analytical Methods for the Characterization of Vegetable Oils. Molecules.

[25] Miklavčič Višnjevec A., Baker P., Charlton A., Preskett D., Peeters K., Tavzes Č., Kramberger K., Schwarzkopf M. (2020). Developing an Olive Biorefinery in Slovenia: Analysis of Phenolic Compounds Found in Olive Mill Pomace and Wastewater. Molecules.

[26] Sales C., Portolés T., Johnsen L.G., Danielsen M., Beltran J. (2019). Olive oil quality classification and measurement of its organoleptic attributes by untargeted GC–MS and multivariate statistical-based approach. Food Chem..

[27] Aparicio-Ruiz R., Casadei E., Ortiz-Romero C., García-González D.L., Servili M., Selvaggini R., Lacoste F., Escobessa J., Vichi S., Quintanilla-Casas B. (2023). Method for the analysis of volatile compounds in virgin olive oil by SPME-GC-MS or SPME-GC-FID. MethodsX.

[28] Apetrei C., Ghasemi-Varnamkhasti M., Mirela Apetrei I. (2016). Olive Oil and Combined Electronic Nose and Tongue. Electronic Noses and Tongues in Food Science.

[29] Souayah F., Rodrigues N., Veloso A.C.A., Dias L.G., Pereira J.A., Oueslati S., Peres A.M. (2017). Discrimination of Olive Oil by Cultivar, Geographical Origin and Quality Using Potentiometric Electronic Tongue Fingerprints. J. Am. Oil Chem. Soc..

[30] Apetrei C. (2012). Novel method based on polypyrrole-modified sensors and emulsions for the evaluation of bitterness in extra virgin olive oils. Food Res. Int..

[31] Barea-Ramos E., Lozano J.D., Aliaño-González M.J., Domínguez Pérez I., Romero-González R., Martín-Tornero E., Diego Barea-Ramos J., Lozano J., Durán-Merás I., Martín-Vertedor D. (2023). E-Nose Quality Evaluation of Extra Virgin Olive Oil Stored in Different Containers. Chemosensors.

[32] Munteanu I.G., Apetrei C. (2023). Classification and Antioxidant Activity Evaluation of Edible Oils by Using Nanomaterial-Based Electrochemical Sensors. Int. J. Mol. Sci..

[33] Pizarro M.L., Becerra M., Sayago A., Beltrán M., Beltrán R. (2013). Comparison of Different Extraction Methods to Determine Phenolic Compounds in Virgin Olive Oil. Food Anal. Methods.

[34] Aluyor E.O., Ozigagu C.E., Oboh O.I. (2009). Aluyor Chromatographic analysis of vegetable oils: A review. Sci. Res. Essay.

[35] Hrncirik K., Fritsche S. (2004). Comparability and reliability of different techniques for the determination of phenolic compounds in virgin olive oil. Eur. J. Lipid Sci. Technol..

[36] Carrasco-Pancorbo A., Cerretani L., Bendini A., Segura-Carretero A., Gallina-Toschi T., Fernández-Gutiérrez A. (2005). Analytical determination of polyphenols in olive oils. J. Sep. Sci..

[37] Alves E., Domingues M.R.M., Domingues P. (2018). Polar Lipids from Olives and Olive Oil: A Review on Their Identification, Significance and Potential Biotechnological Applications. Foods.

[38] Nichitoi M.M., Josceanu A.M., Isopescu R.D., Isopencu G.O., Geana E.I., Ciucure C.T., Lavric V. (2021). Polyphenolics profile effects upon the antioxidant and antimicrobial activity of propolis extracts. Sci. Rep..

[39] Hohrenk L.L., Itzel F., Baetz N., Tuerk J., Vosough M., Schmidt T.C. (2020). Comparison of Software Tools for Liquid Chromatography-High-Resolution Mass Spectrometry Data Processing in Nontarget Screening of Environmental Samples. Anal. Chem..

[40] Bendini A., Cerretani L., Carrasco-Pancorbo A., Gómez-Caravaca A.M., Segura-Carretero A., Fernández-Gutiérrez A., Lercker G. (2007). Phenolic Molecules in Virgin Olive Oils: A Survey of Their Sensory Properties, Health Effects, Antioxidant Activity and Analytical Methods. An Overview of the Last Decade Alessandra. Molecules.

[41] Kabaran S. (2018). Olive Oil: Antioxidant Compounds and Their Potential Effects over Health. Functional Foods.

[42] Ajal E.A., Chaji S., Moussafir S., Nejjari R., Soulaymani A., Bajoub A. (2021). Virgin Olive Oil Phenolic Compounds: Insights on Their Occurrence, Health-Promoting Properties and Bioavailability. Olive Oil-New Perspectives and Application.

[43] Becerra-Herrera M., Vélez-Martín A., Ramos-Merchante A., Richter P., Beltrán R., Sayago A. (2018). Characterization and evaluation of phenolic profiles and color as potential discriminating features among Spanish extra virgin olive oils with protected designation of origin. Food Chem..

[44] Arslan D., Karabekir Y., Schreiner M. (2013). Variations of phenolic compounds, fatty acids and some qualitative characteristics of Sarıulak olive oil as induced by growing area. Food Res. Int..

[45] Bubola K.B., Lukić M., Novoselić A., Krapac M., Lukić I. (2020). Olive Fruit Refrigeration during Prolonged Storage Preserves the Quality of Virgin Olive Oil Extracted Therefrom. Foods.

[46] Di Stefano V., Melilli M.G. (2019). Effect of storage on quality parameters and phenolic content of Italian extra-virgin olive oils. Nat. Prod. Res..

[47] Faghim J., Mohamed M.B., Bagues M., Nagaz K., Triki T., Guasmi F. (2021). Irrigation effects on phenolic profile and extra virgin olive oil quality of “Chemlali” variety grown in South Tunisia. S. Afr. J. Bot..

[48] Lechhab T., Salmoun F., Lechhab W., El Majdoub Y.O., Russo M., Camillo M.R.T., Trovato E., Dugo P., Mondello L., Cacciola F. (2021). Determination of bioactive compounds in extra virgin olive oils from 19 Moroccan areas using liquid chromatography coupled to mass spectrometry: A study over two successive years. Eur. Food Res. Technol..

[49] Klisović D., Koprivnjak O., Novoselić A., Pleadin J., Lešić T., Brkić Bubola K. (2022). Compositional Changes in the Extra Virgin Olive Oil Used as a Medium for Cheese Preservation. Foods.

[50] Sun W., Frost B., Liu J. (2017). Oleuropein, unexpected benefits!. Oncotarget.

[51] Dauber C., Carreras T., González L., Gámbaro A., Valdés A., Ibañez E., Vieitez I. (2022). Characterization and incorporation of extracts from olive leaves obtained through maceration and supercritical extraction in Canola oil: Oxidative stability evaluation. LWT.

