# Supplementary material for: Discrimination of Olive Oil and Extra-Virgin Olive Oil from Other Vegetable Oils by Targeted and Untargeted HRMS Profiling of Phenolic and Triterpenic Compounds Combined with Chemometrics
Source: Int J Mol Sci. 2023 Mar 10;24(6):5292. doi: 10.3390/ijms24065292 (PMC10049382; doi:10.3390/ijms24065292)
Supplement: Supplementary file 1 [file ijms-24-05292-s001.zip › ijms-2241253-SI.pdf]

International Journal of *Molecular Sciences*

MDPI

Supplementary materials

Discrimination of Olive Oil and Extra-Virgin Olive Oil from Other Vegetable Oils by Targeted and Untargeted HRMS Profiling of Phenolic and Triterpenic Compounds Combined with Chemometrics

Elisabeta-Irina Geana [1], Corina Teodora Ciucure [1], Irina Mirela Apetrei [2], Maria Lisa Clodoveo [3] and Constantin Apetrei [4,*]

1 National Research and Development Institute for Cryogenics and Isotopic Technologies—ICSI, Rm. Valcea, 240050 Râmnicu Vâlcea, Romania
2 Department of Pharmaceutical Sciences, Medical and Pharmaceutical Research Center, Faculty of Medicine and Pharmacy, "Dunarea de Jos" University of Galati, 800008 Galati, Romania
3 Interdisciplinary Department of Medicine, University Aldo Moro Bari, 70125 Bari, Italy
4 Department of Chemistry, Physics and Environment, "Dunarea de Jos" University of Galati, 800008 Galati, Romania
* Correspondence: apetreic@ugal.ro; Tel.: +40-727-580914

Table S1. Phenolic compounds from different oil types (EVOO* - authentic extra virgin oils, EVOO – commercially available extra virgin oils, VOO – commercially available virgin olive oil, SF – sunflower oils, other vegetable oils including walnut, grape seed, pumpkin, linseed, soybean, sesame, palm, hemp and coconut oils) expressed as μg/g of oil.

Compounds	EVOO*	EVOO	VOO	SF	Vegetable oils
3,4-Dihydroxybenzoic acid	0.04±0.03 (n.d.-0.12)	0.03±0.02 (0.01-0.06)	0.03±0.02 (n.d. -0.06)	0.01±0.02 (n.d. -0.04)	0.01±0.00 (n.d. -0.02)
4-Hydroxybenzoic acid	0.07±0.03 (n.d.-0.13)	0.08±0.03 (0.04-0.12)	0.06±0.04 (n.d. -0.13)	0.03±0.05 (n.d. -0.12)	0.07±0.13 (n.d. -0.38)
t-Ferulic acid	0.11±0.07 (n.d.-0.30)	0.10±0.05 (0.05-0.18)	0.09±0.08 (0.01-0.24)	0.09±0.18 (n.d. -0.42)	0.22±0.27 (0.01-1.01)
Chlorogenic acid	n.d. (0.00-0.01)	n.d. (n.d. -0.01)	0.01±0.02 (n.d. -0.09)	0.02±0.03 (n.d. -0.08)	0.01±0.01 (n.d. -0.05)
Cinnamic acid	2.81±1.48 (n.d.-5.08)	2.78±0.83 (1.92-3.82)	2.34±1.42 (0.02-4.83)	0.33±0.66 (0.01-1.52)	0.10±0.11 (0.01-0.37)
p-Coumaric acid	0.31±0.20 (n.d.-0.81)	0.46±0.08 (0.37-0.54)	0.33±0.20 (0.01-0.71)	0.02±0.04 (0.01-0.09)	0.04±0.05 (0.01-0.18)
Ellagic acid	0.04±0.05 (n.d.-0.18)	0.02±0.01 (n.d. -0.04)	0.04±0.05 (n.d. -0.16)	0.06±0.06 (0.01-0.16)	0.17±0.21 (0.02-0.73)
Abscisic acid	0.01±0.01 (n.d.-0.04)	0.01±0.01 (n.d. -0.02)	0.01±0.01 (n.d. -0.03)	0.05±0.11 (0.00-0.24)	0.03±0.11 (n.d. -0.42)
Rutin	n.d. (n.d. -0.01)	n.d.	0.01±0.01 (n.d. -0.03)	0.01±0.00 (0.01-0.02)	0.02±0.00 (0.01-0.03)
Quercetin	n.d. (n.d. -0.02)	0.01±0.02 (n.d. -0.04)	0.01±0.02 (n.d. -0.06)	0.00±0.01 (n.d. -0.01)	0.01±0.01 (n.d. -0.05)
Isorhamnetin	0.01±0.01 (n.d. -0.04)	0.01±0.01 (n.d. -0.02)	0.01±0.01 (n.d. -0.05)	0.01±0.02 (n.d. -0.05)	0.02±0.01 (n.d. -0.04)
Kaempferol	n.d. (n.d. -0.01)	n.d.	0.00±0.00 (n.d. -0.02)	0.00±0.00 (n.d. -0.01)	0.02±0.02 (n.d. -0.05)
Apigenin	1.58±1.60 (n.d.-6.49)	1.03±0.34 (0.74-1.58)	1.03±0.67 (0.01-1.95)	0.01±0.01 (n.d.-0.02)	0.02±0.01 (0.01-0.05)
Pinocembrin	0.02±0.03 (n.d.-0.11)	0.11±0.05 (0.02-0.16)	0.07±0.09 (0.01-0.38)	0.01±0.02 (0.01-0.04)	0.01±0.00 (n.d. -0.02)
Chrysin	0.01±0.00 (n.d. -0.02)	0.01±0.00 (n.d. -0.01)	0.01±0.00 (0.00-0.02)	0.01±0.00 (n.d. -0.01)	0.01±0.01 (n.d. -0.04)
Hydroxytyrosol	5.44±8.11 (0.01-24.58)	3.66±4.61 (0.01-10.72)	0.87±1.57 (n.d.-5.38)	n.d.	n.d.
Tyrosol	0.53±0.88 (n.d.-2.55)	1.19±1.81) n.d.-4.36	1.89±2.73 (n.d.-10.39)	3.24±2.97 (0.39-7.32)	5.19±4.09 (n.d.-12.76)

Trigoneline	11.87±7.24 (0.83-22.51)	14.78±11.50 (5.77-34.06)	9.70±6.44 (2.07-26.99)	4.78±5.09 (1.01-13.58)	12.08±17.44 (n.d.-65.13)
Verbascoside	0.41±0.13 (0.31-0.76)	0.36±0.07 (0.30-0.47)	0.34±0.10 (0.29-0.77)	0.18±0.17 (n.d.-0.31)	0.28±0.08 (n.d.-0.31)
Oleuropein	9.75±5.56 (0.81-18.91)	14.04±7.44 (0.30-21.81)	10.44±7.94 (0.81-33.00)	4.63±4.93 (2.01-33.00)	7.66±8.18 (n.d.-32.10)
Maslinic_acid	2.58±1.55 (0.76-5.84)	4.31±5.59 (1.62-14.31)	5.52±5.32 (0.24-18.73)	0.11±0.08 (n.d.-0.22)	0.49±1.20 (n.d.-4.53)
Oleanolic_acid	0.17±0.17 (0.03-0.66)	2.05±2.30 (0.69-6.15)	2.96±4.53 (0.07-16.07)	0.65±0.25 (0.25-0.91)	1.28±4.11 (n.d.-15.45)

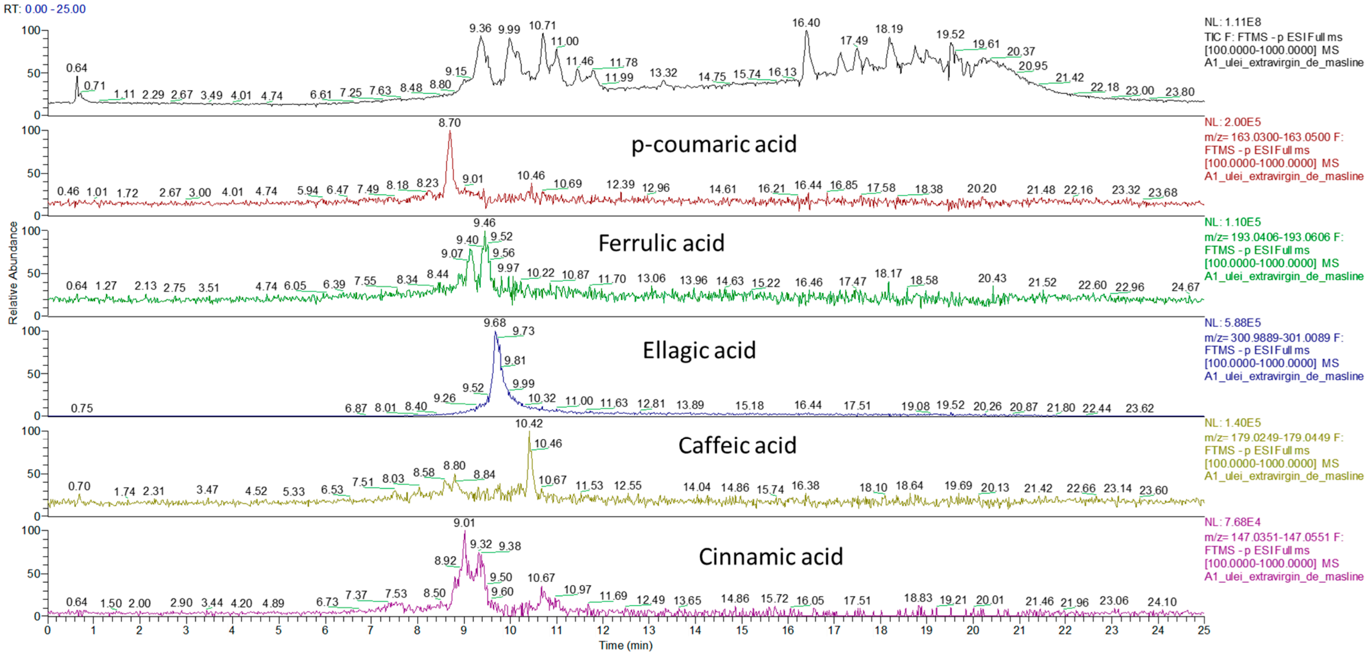

(A)

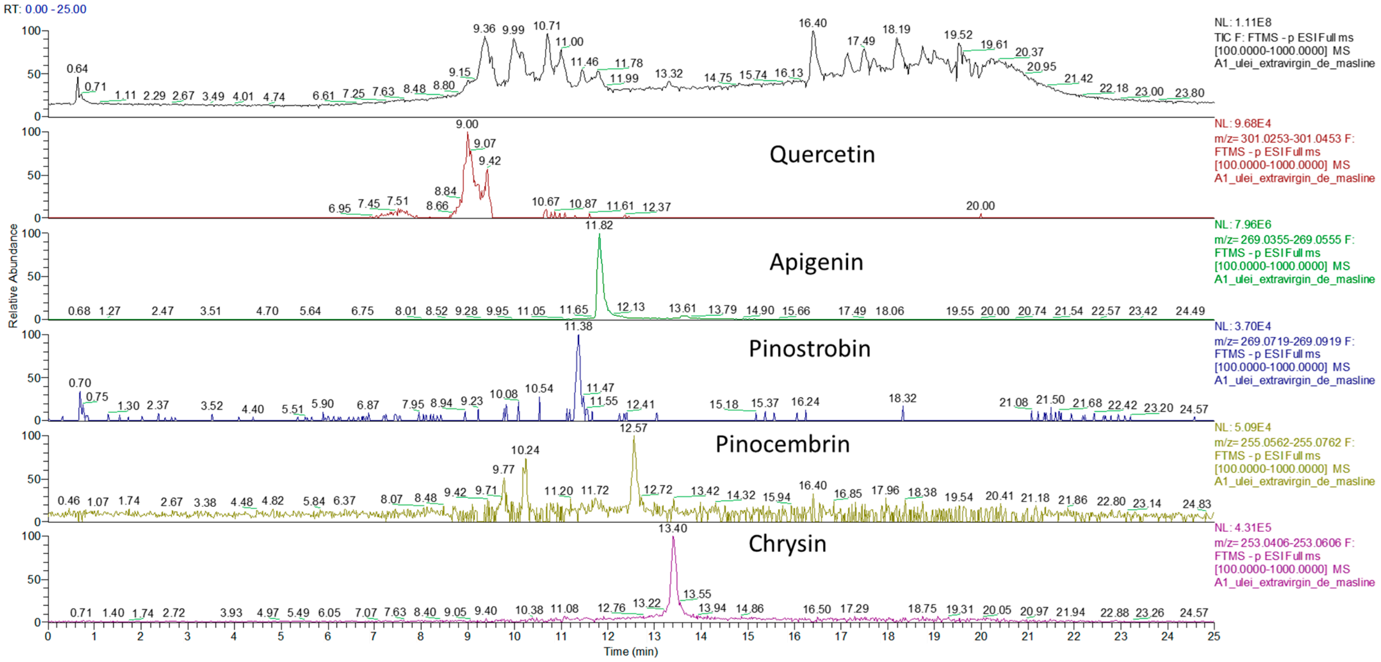

(B)

Figure S1. TIC and the extracted chromatograms of the minor phenolic compounds quantified in liquid extract of extra virgin olive oil (the chromatograms were extracted from TIC using a 5 ppm mass accuracy window; negative ion mode, full scan, base peak in the range 75-1000 m/z): (A) phenolic acids, (B) flavonoids

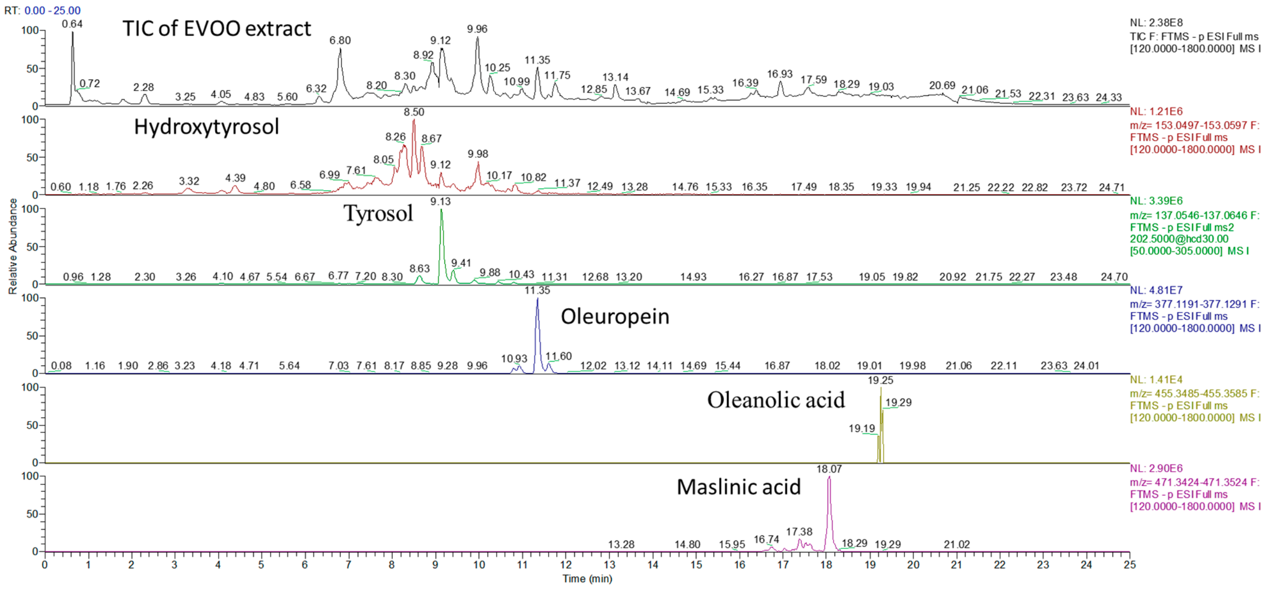

Figure S2. TIC and the extracted chromatograms of the phenolic and triterpenic compounds quantified in liquid extract of extra virgin olive oil (the chromatograms were extracted from TIC using a 5 ppm mass accuracy window; negative ion mode, full scan, base peak in the range 75-1000 m/z).

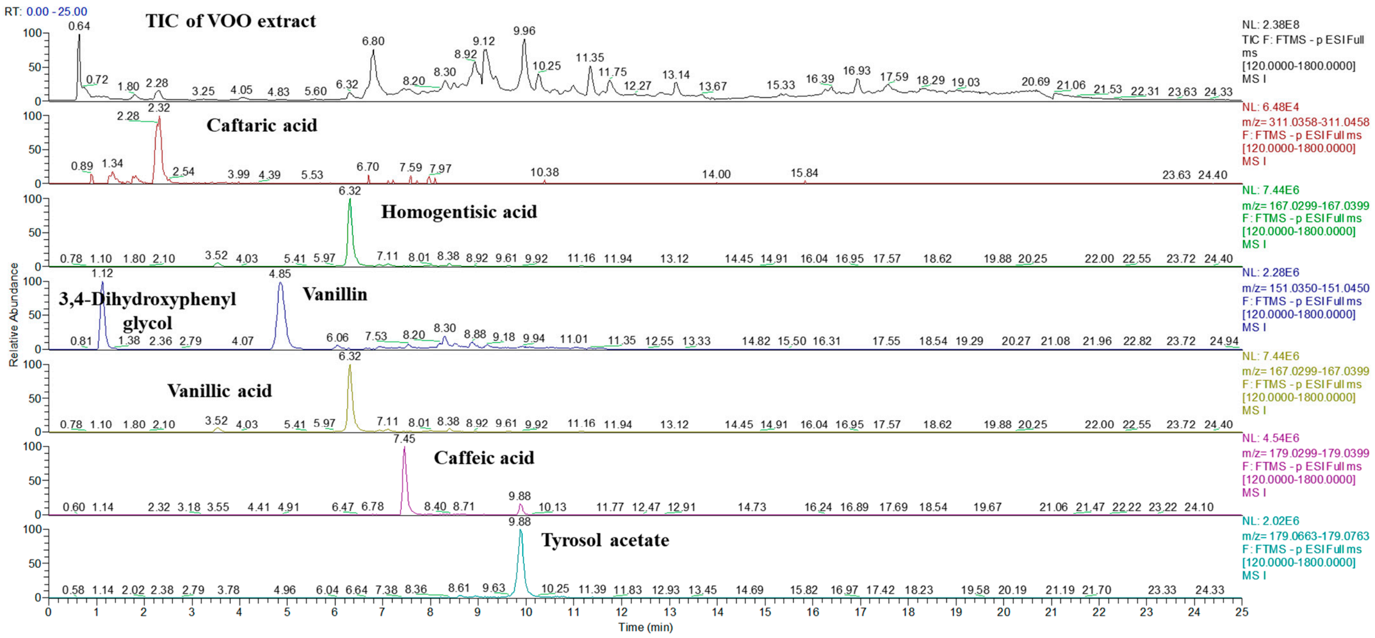

(A)

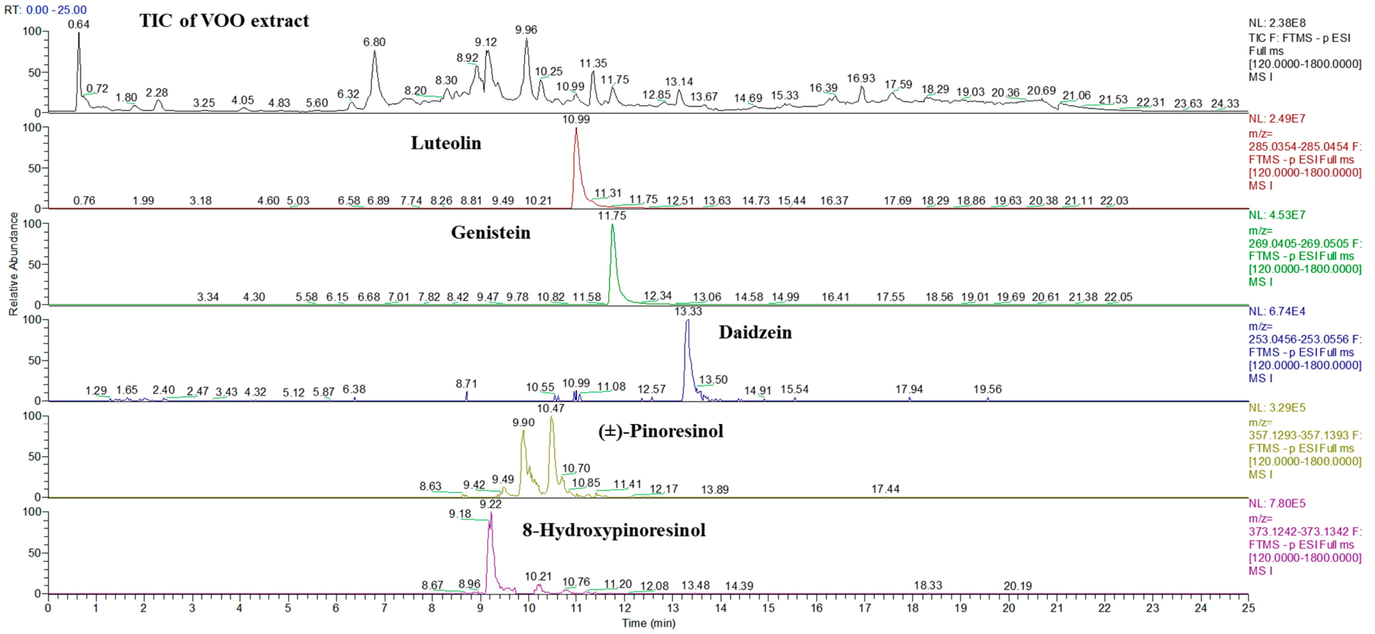

(B)

Figure S3. TIC and the extracted chromatograms of the specific compounds identified in VOO extract (the chromatograms were extracted from TIC using a 5 ppm mass accuracy window; negative ion mode, full scan, base peak in the range 75-1000 m/z): (A) simple phenols & derivatives and (B) flavonoids and lignans.

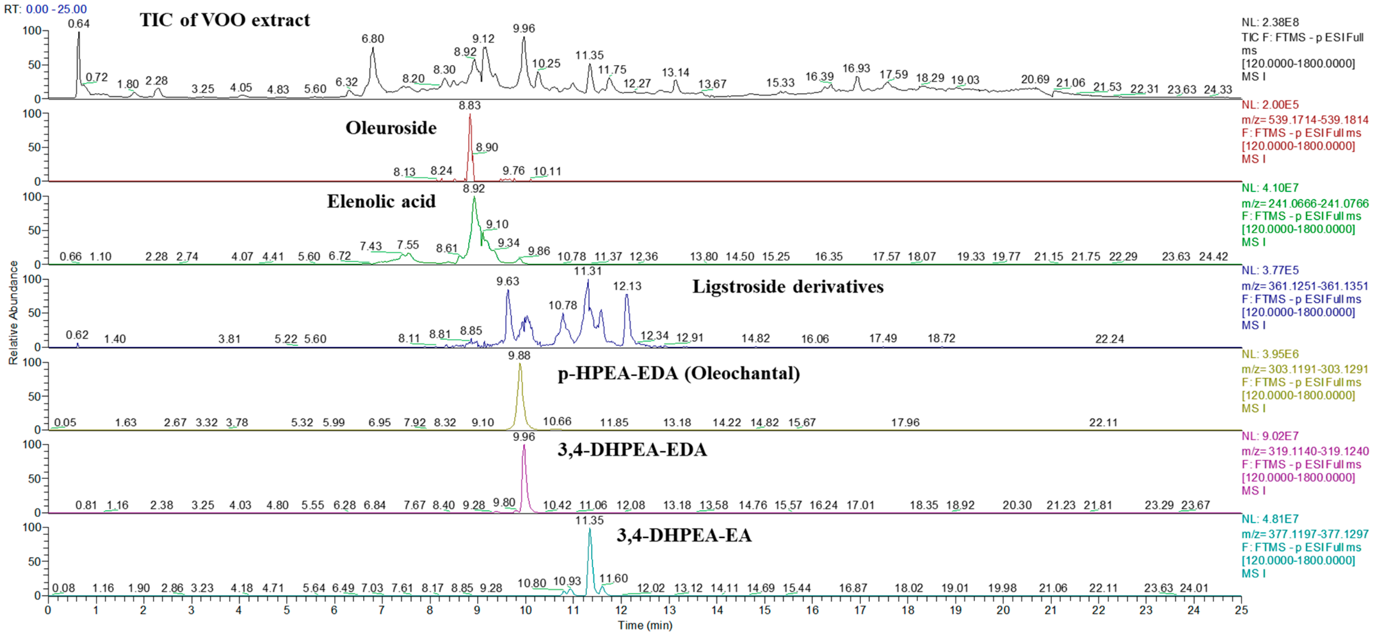

Figure S4. TIC and the extracted chromatograms of the secoiridoids and derivatives identified in VOO extract (the chromatograms were extracted from TIC using a 5 ppm mass accuracy window; negative ion mode, full scan, base peak in the range 75-1000 m/z).
